# Minimizing VPD Fluctuations Maintains Higher Stomatal Conductance and Photosynthesis, Resulting in Improvement of Plant Growth in Lettuce

**DOI:** 10.3389/fpls.2021.646144

**Published:** 2021-04-01

**Authors:** Takayasu Inoue, Motoo Sunaga, Mutsuhiro Ito, Qu Yuchen, Yoriko Matsushima, Kazuma Sakoda, Wataru Yamori

**Affiliations:** ^1^Fuji Chemical Co., Ltd., Gifu, Japan; ^2^Fuji Silysia Chemical Co., Ltd., Gifu, Japan; ^3^Institute for Sustainable Agro-Ecosystem Services, The University of Tokyo, Nishitokyo, Japan

**Keywords:** photosynthesis, VPD, lettuce, rockwool, relative air humidity, stomatal conductance

## Abstract

Vapor pressure deficit (VPD) is considered to be one of the major environmental factors influencing stomatal functions and photosynthesis, as well as plant growth in crop and horticultural plants. In the greenhouse cultivation, air temperature and relative air humidity are regulated by switching on/off the evaporative systems and opening/closing the roof windows, which causes VPD fluctuation. However, it remains unclear how VPD fluctuation affects photosynthetic and growth performance in plants. Here, we examined the effects of the VPD fluctuation on the photosynthetic and growth characteristics in lettuce (*Lactuca sativa* L.). The parameters for gas exchange and chlorophyll fluorescence and biomass production were evaluated under the conditions of drastic (1.63 kPa for 6 min and 0.63 for 3 min) or moderate (1.32 kPa for 7 min and 0.86 kPa for 3 min) VPD fluctuation. The drastic VPD fluctuation induced gradual decrease in stomatal conductance and thus CO_2_ assimilation rate during the measurements, while moderate VPD fluctuation caused no reduction of these parameters. Furthermore, data showed moderate VPD fluctuation maintained leaf expansion and the efficiency of CO_2_ diffusion across leaf surface, resulting in enhanced plant growth compared with drastic VPD fluctuation. Taken together, fine regulation of VPD can be crucial for better plant growth by maintaining the photosynthetic performance in lettuce. The present work demonstrates the importance of VPD control during plant cultivation in plant factories and greenhouses.

## Introduction

Natural resource availability has been a limitation of agricultural industry throughout human history; agricultural production has been greatly threatened by water and nutrition shortage and insufficient available land for centuries. In recent years, the development of agricultural technology has enabled a crop cultivation under indoor environments, and the indoor agriculture could protect crops from harmful environments (Kozai et al., 2015). The current trend in greenhouse cultivation is to extend the crop growing season in order to maximize the equipment operation, elongate the exporting season, and increase the annual yield per unit area, resulting in the profitability improvement.

One major advance for greenhouse and indoor agriculture is water controlling. Plants in greenhouse or similar facilities are less likely to suffer from air water deficit compared with open field. In general, most plants would grow well at vapor pressure deficit (VPD) between 0.5 and 0.8 kPa (Bakker, 1991). The reduction of transpiration rate at high VPD is observed in most crop species (> 2.0 kPa; Gholipoor et al., 2010; Zaman-Allah et al., 2011). Guard cells are vulnerable to turgor loss under high VPD. When the water flux into the stem is too low to meet the high transpiration rate at the leaf, it will eventually cause the closing of stomata. Thus, the stomatal closure decreases the conductance of gas diffusion via stomata, or, stomatal conductance (Chaves, 1991; Ort et al., 1994; Chaves et al., 2003; Flexas et al., 2004), resulting in a decrease in CO_2_ assimilation rate (Sinclair et al., 2017). Atmosphere water deficit can suppress the photosynthetic performance also by directly impairing metabolic activities including the enzyme activity of Calvin-Benson cycles (Farquhar et al., 1989) and leads to the loss of biomass production throughout the crop growing period (Tibbitts, 1979; Grange and Hand, 1987; Bakker, 1991; Marsden et al., 1996; Leuschner, 2002; Codarin et al., 2006).

Evaporative systems for cooling and humidifying greenhouses have been developed to provide the ideal growing conditions in a greenhouse (Brandon et al., 2016). Establishment of the appropriate combination of air and water supply would depend on the environmental conditions such as radiation from the sun, ambient temperature and relative air humidity, and it would be essential for maintaining the desired conditions for the plant cultivation in the greenhouse. The main evaporative cooling methods used today are a fan-and-pad system (Davies, 2005) and a fogging system (Sase et al., 2006; Hayashi et al., 2007; Perdigones et al., 2008; Lu et al., 2015). The performance of the fogging system is superior to that of the fan-and-pad system, regarding the uniform distribution of temperature and relative air humidity in the greenhouse (Arbel et al., 2003; Abdel-Ghany and Kozai, 2006; Toida et al., 2006). Research on the plant responses to fogging conditions by the fogging system demonstrated that a fogging system can efficiently improve plant growth (Katsoulas et al., 2001; Leyva et al., 2013).

VPD depends on both temperature and relative air humidity. Evaporative systems mentioned above are used in different situations and in various forms (Brandon et al., 2016; Aljubury and Ridha, 2017), however, most of such instruments are simply controlled by switching on/off, which would cause fluctuation in air humidity due to its binary controlling manner. Therefore, high VPD could be observed during the day even in greenhouse environments (Harmanto et al., 2005; Lu et al., 2015; Zhang et al., 2015). In addition, the temperature in greenhouses often exceed 30°C during midday of sunny winter days in Asian countries. Growers must open the roof windows during this period to lower the temperature inside the greenhouse. The air exchange between the outside and inside of the greenhouse would increase VPD because of the low air humidity in cold seasons (Lu et al., 2015). Thus, VPD can be drastically affected by the on/off switching of evaporative systems and the opening/closing of roof windows. However, to our knowledge, there have been no report studying the effects of a fluctuating VPD condition on the photosynthetic and growth performance in plants. Understanding physiological mechanisms underlying the effect of fluctuating VPD condition on the plant growth would be important for efficient agricultural production in greenhouse with highly controlled environmental conditions. This study was aimed to characterize the effect of the VPD fluctuation on the photosynthetic and growth characteristics in lettuce (*Luctica sativa* L.), the most common vegetable cultivated in greenhouses.

## Materials and Methods

### Plant Materials and Growth Conditions

Average, amplitude and cycles of VPD were set according to the values typically monitored in green houses (Garcia et al., 2011; Lu et al., 2015; Zhang et al., 2015). To evaluate the long-term effects of the VPD conditions, romaine lettuce (*Lactuca sativa* L. var. Romana; Takii Seed Co., Kyoto, Japan) were sown in rockwool in an environmentally controlled growth chamber (NK Systems, Japan) at a PPFD of 200 μmol photons m^−2^ s^−1^, a 16 h photoperiod, a CO_2_ concentration of 400 μmol mol^−1^ and two different VPD conditions: moderately fluctuating VPD condition in which a cycle of high VPD (1.32 kPa = relative air humidity of 55%) for 7 min and low VPD (0.86 kPa = relative air humidity of 72%) for 3 min was repeated for 24 h before measurement, and drastically fluctuating VPD condition in which a cycle of high VPD (1.63 kPa = relative air humidity of 42%) for 6 min and low VPD (0.63 kPa = relative air humidity of 80%) for 3 min was repeated for 24 h before measurement. The averages of daily VPD, relative air humidity and temperature were similar between treatments (1.03 ± 0.20 kPa, 65.5 ± 6.9% and 24.0 ± 0.3°C for moderate fluctuating VPD condition, 1.04 ± 0.35 kPa, 64.4 ± 13.7% and 24.0 ± 0.8°C for drastic fluctuating VPD condition). The plants were supplied with sufficient nutrient solution to avoid drought stress. Each rockwool was given 100 ml of nutrient solution at 1/1000 strength (HYPONeX, N:P:K, 6:10:5, Hyponex Japan, Osaka, Japan) everyday.

### Analyses of Chlorophyll Fluorescence, P700 and Gas Exchange Measurement

Chlorophyll fluorescence, P700 redox state and gas exchange were measured simultaneously during a 16 h photoperiod using a Dual-PAM-100 and a GFS-3000 measuring systems (Walz, Effeltrich, Germany) in uppermost, fully expanded new leaves of 3-week-old plants grown under moderately fluctuating VPD condition, as described in Yamori et al. (2015, 2016). After leaves were dark-adapted for 30 min, a saturating pulse was applied to obtain the maximum fluorescence and the maximum change in P700. For measurements of photosynthetic parameters, the leaf was firstly allowed to equilibrate at 0.63 kPa or 0.48 kPa of VPD at a CO_2_ concentration of 400 μmol mol^−1^ and a PPFD of 200 μmol photons m^−2^ s^−1^ for at least 30 min. A CO_2_ assimilation rate and stomatal conductance were measured every 1 min in the environmentally controlled chamber of portable photosynthesis system under two different VPD conditions: moderately fluctuating VPD, in which a cycle of VPD of 0.63 kPa (=relative air humidity of 80%) for 5 min, VPD of 1.27 kPa (=relative air humidity of 60%) for 4 min and then VPD of 0.95 kPa (=relative air humidity of 70%) for 3 min was repeated for 400 min (maximum amplitude: VPD=0.64kPa, relative air humidity=20%), and drastically fluctuating VPD, in which a cycle of VPD of 0.48 kPa (=relative air humidity of 85%) for 3 min, VPD of 1.74 kPa (=relative air humidity of 45%) for 3 min and then VPD of 0.95 kPa (=relative air humidity of 70%) for 3 min was repeated for 400 min (maximum amplitude: VPD = 1.26, relative air humidity = 40%).

The quantum yield of photosystem I (ΦPS I) was calculated from the complementary PS I quantum yields of non-photochemical energy dissipation, Y(ND) and Y(NA): Y(I) = 1–Y(ND)–Y(NA). The quantum yield of photosystem II [ΦPS II], photochemical quenching [qP] and the fraction of PS II centers in the open state (with plastoquinone oxidized) [qL] were calculated. The electron transport rate (ETR) was calculated as ETR I (or ETR II) = 0.5 × 0.84 × φPS I (or φPS II), where 0.5 is the fraction of absorbed light reaching PS I or PS II, and 0.84 is the leaf absorptance (Genty et al., 1989).

**Figure 1:**
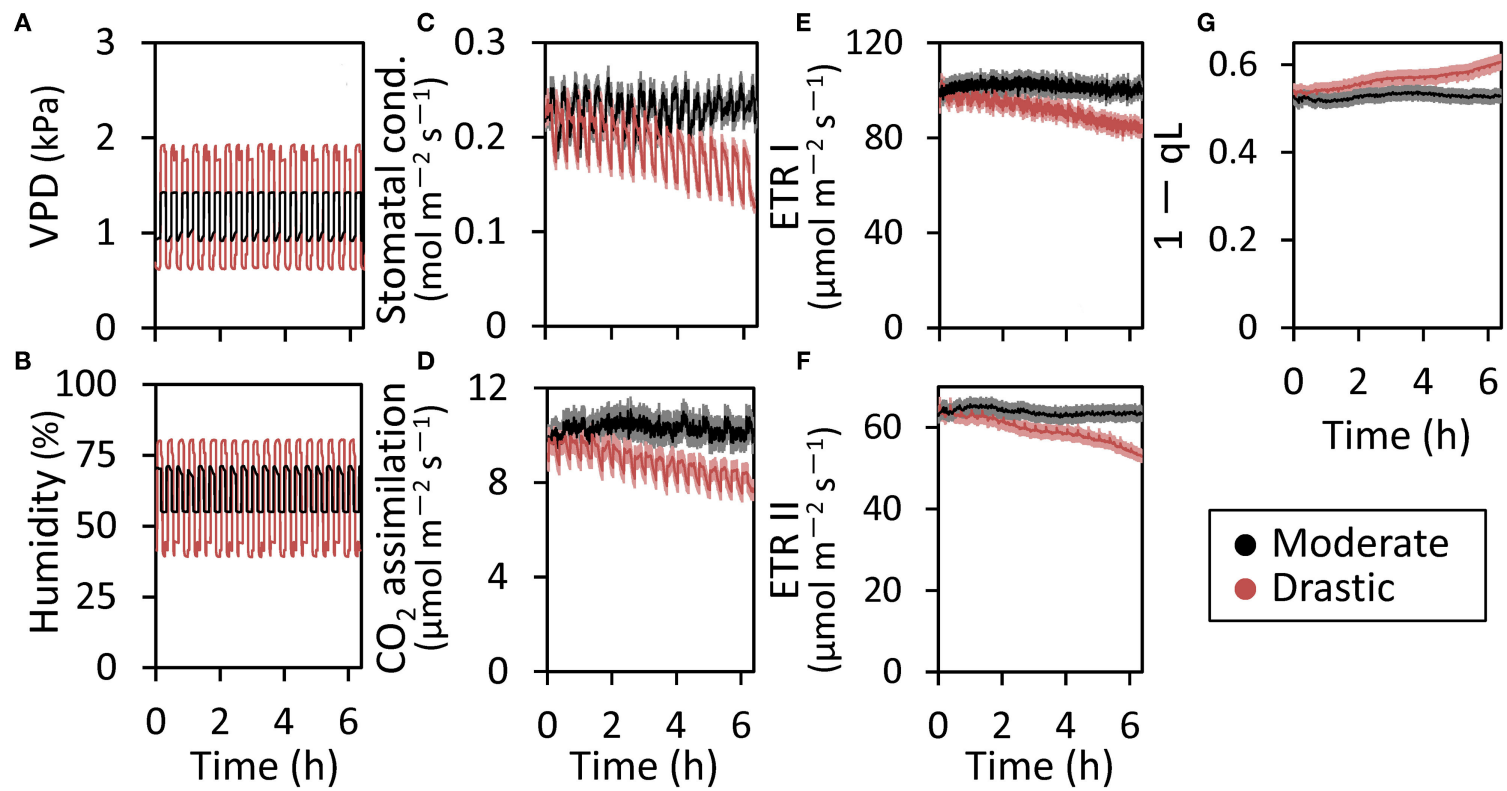
Responses of photosynthetic parameters to moderate and drastic VPD fluctuation in lettuce grown under moderately fluctuating VPD condition. Both VPD **(A)** and relative air humidity **(B)** in the environmentally controlled chamber of portable photosynthesis system were shown. The stomatal conductance **(C)**, CO_2_ assimilation rate **(D)**, electron transport rate through PSI (ETR I) **(E)** and through PSII (ETR II) **(F)**, and the redox state of the plastoquinone pool (1-qL) **(G)** were monitored at a CO_2_ concentration of 400 μmol mol^−1^ and a PPFD of 200 μmol photons m^−2^ s^−1^ under either moderately or drastically fluctuating VPD conditions for 6 h. The average leaf temperature over the entire period was 25.5 ± 0.32°C for the moderate VPD treatment and 25.8 ± 0.31°C for the drastic VPD treatment. The data are the means ± standard errors of six biological replicates.

The maximum level of the P700 signal (Pm, full oxidation of P700) and the maximum quantum yield of PS II (Fv/Fm) in the dark was analyzed after the measurements of photosynthesis under moderate or drastic VPD fluctuation as shown in Figure 1. The leaves were placed in a temperature-controlled chamber at a CO_2_ concentration of 400 μmol mol^−1^ and leaf temperature at 25°C in a Dual-PAM-100 and a GFS-3000 measuring system (Walz, Effeltrich, Germany), and exposed to (1) moderate fluctuating VPD or (2) drastic fluctuating VPD for 5 h at 200 μmol photons m^−2^ s^−1^. The Pm and Fv/Fm after dark incubation for 15 min were measured before and after the light treatments (Yamori et al., 2016).

### Analysis of Gas Exchange Under Different Duration and Frequency of VPD

We also measured a CO_2_ assimilation rate, stomatal conductance, transpiration rate, and intercellular CO_2_ concentration under different duration and frequency of VPD fluctuation cycles using LI-6800 (Li-Cor, Lincoln, NE, USA) using the 3-week-old lettuce grown under moderately fluctuating VPD condition. For measurements of photosynthesis parameters, the leaf was first allowed to equilibrate at 1.0 kPa of VPD at a PPFD of 200 μmol photons m^−2^ s^−1^ for at least 30 min, and the photosynthetic parameters were recorded every 4 min under various VPD fluctuations, throughout the 400 min period. Three types of VPD condition were generated in the environmentally controlled chamber of portable photosynthesis system: constant VPD of 0.95 kPa (=relative air humidity of 70%), rapidly fluctuating VPD condition in which a cycle of VPD of 0.48 kPa (=relative air humidity of 85%) for 3 min, VPD of 1.74 kPa (=relative air humidity of 45%) for 3 min and then VPD of 0.95 kPa (=relative air humidity of 70%) for 3 min was repeated for 400 min (maximum amplitude: VPD = 1.26, relative air humidity = 40%) and slowly fluctuating VPD condition in which a cycle of VPD of 0.63 kPa (=relative air humidity of 80%) for 5 min, VPD of 1.27 kPa (=relative air humidity of 60%) for 4 min and then VPD of 0.95 kPa (=relative air humidity of 70%) for 3 min was repeated for 400 min (maximum amplitude: VPD = 0.64 kPa, relative air humidity = 20%). The average of VPD was similar between treatments and showed ~0.92 kPa (=relative air humidity of 71%). VPD values were calculated according to Buck (1981); VPD = 0.611e [17.502 × Temperature/(Temperature+240.97)] × (1–Relative Air Humidity).

### Plant Growth Analysis

The plants cultivated in the environmentally controlled growth chamber were harvested every week after transplanting and shoot fresh weights and total leaf area were measured. The shoot samples were dried under 80°C in an oven for one week and then weighted. Leaf area was measured using a LI-3000 leaf area meter (Li-Cor, Lincoln, NE, USA). Leaf mass per area (LMA) was measured as dry weight per unit leaf area. The experiment of plant growth analysis was repeated twice, switching the treatments between chambers. Number of leaves larger than 0.3 mm were counted by eye on 3rd week.

### Chlorophyll and Anthocyanin Contents

The contents of chlorophyll and anthocyanin were quantified at fully expanded leaves of plants grown under two fluctuating VPD conditions for three weeks, using a spectrophotometric method as described in Porra et al. (1989) and ACM-200plus equipment (Opti-Sciences, Inc., USA), respectively.

### Statistical Analysis

The data represent the means for three replicate samples of two independent experiment. Data are presented as means ± SE. Analysis of Student *t*-test was performed in the SPSS statistical software (SPSS, Chicago, IL). Differences were considered significant at *P* < 0.05.

## Results

### Effects of the Amplitude of the VPD Fluctuation on the Photosynthetic Characteristics

The fluctuating VPD condition in the environmentally controlled chamber of portable photosynthesis system induced the fluctuation of a stomatal conductance, CO_2_ assimilation rate, ETR I and ETR II, although the amplitude of the fluctuation was much larger in the stomatal conductance and CO_2_ assimilation rate than ETR I and ETR II (Figure 1). All the photosynthetic parameters were maintained under moderate VPD fluctuation throughout the measurements for 6 h (Figure 1). On the other hand, under drastic VPD fluctuation, all the photosynthetic parameters were not affected during the first 1–2 h after the measurements but declined gradually (Figure 1). In addition, drastic VPD fluctuation induced the gradual increase in the plastoquinone pool (1-qL), indicating that the electron transport system would accumulate reducing power (Figure 1G; Baker, 2008).

**Figure 2:**
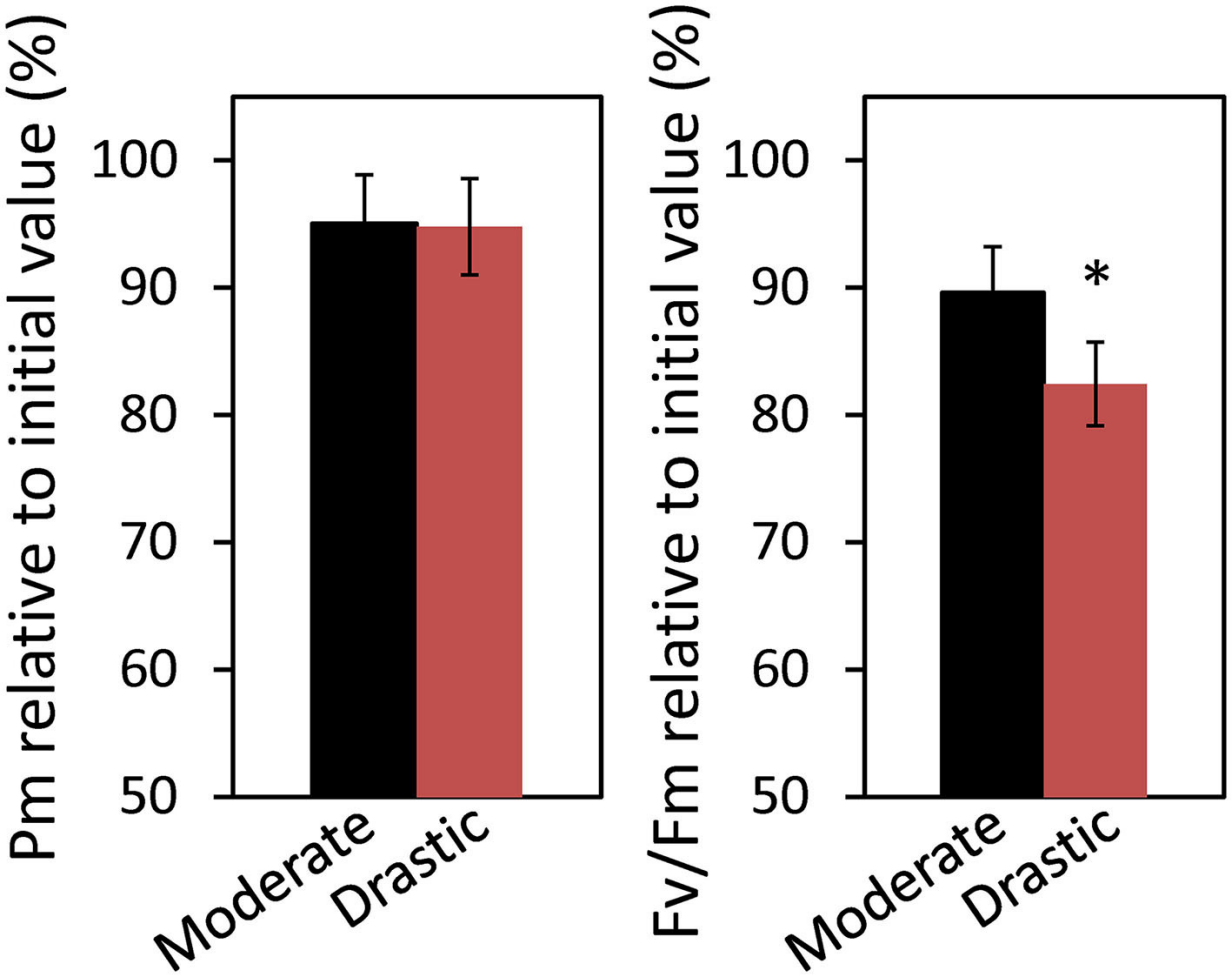
Effect of VPD conditions on alleviation of photoinhibition in lettuce grown under moderately fluctuating VPD condition. The maximum level of the P700 signal of PSI (Pm, full oxidation of P700) and the maximum quantum yield of PSII (Fv/Fm) were measured before and after treatment with moderate VPD or drastic VPD for 6 h, as same in Figure 1. Pm and Fv/Fm relative to the initial values before the treatments are shown. The average of Fv/Fm and Pm at the initial values before the treatments was 0.805 ± 0.003 and 1.15 ± 0.01, respectively. The data are the means ± standard errors of six biological replicates. Significant differences between two different VPD conditions are examined by Student's *t*-test (*P < 0.05).

At the end of the measurement, we also evaluated the extent of photoinhibition both at PSI and PSII caused by the VPD fluctuation (Figure 2). The maximum level of the P700 signal (Pm) under darkness and the maximum quantum yield of PSII (Fv/Fm) were measured before and after moderate and drastic VPD fluctuations. Although the remaining activity of PSI showed no difference between two VPD conditions, that of PSII was significantly lower under drastic VPD fluctuation than moderate VPD fluctuation (Figure 2), suggesting that more photodamage at PSII would be accumulated under drastic VPD fluctuation.

### Effects of the Duration and Frequency of the VPD Fluctuation on the Photosynthetic Characteristics

**Figure 3:**
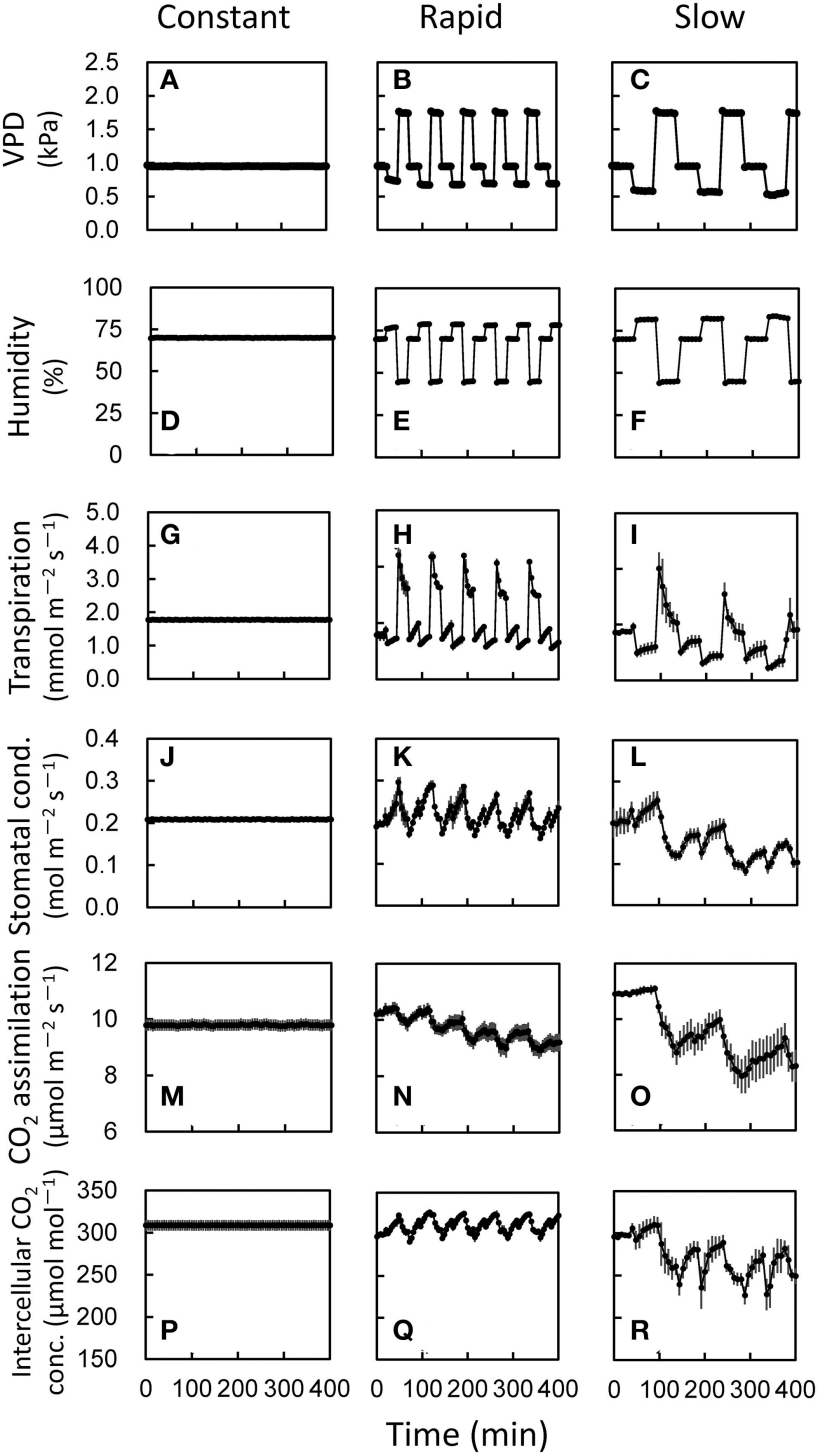
Responses of photosynthetic parameters to several fluctuating VPD conditions in lettuce grown under moderately fluctuating VPD condition. Three different VPD conditions **(A–C)** which corresponds to the relative air humidity **(D–F)** was set in an environmentally controlled growth chamber. The transpiration rate **(G–I)**, stomatal conductance **(J–L)**, CO_2_ assimilation rate **(M–O)**, and intercellular CO_2_ concentration **(P–R)** were measured at a CO_2_ concentration of 400 μmol mol^−1^ and a PPFD of 200 μmol photons m^−2^ s^−1^ under three VPD conditions: (1) constant VPD of 0.95 kPa (=relative air humidity of 70%); (2) rapidly fluctuating VPD, in which VPD of 0.48 kPa (=relative air humidity of 85%) for 3 min, VPD of 1.74 kPa (=relative air humidity of 45%) for 3 min, and VPD of 0.95 kPa (=relative air humidity of 70%) for 3 min; (3) slowly fluctuating VPD, in which VPD of 0.63 kPa (=relative air humidity of 80%) for 5 min, VPD of 1.27 kPa (=relative air humidity of 60%) for 4 min, and VPD of 0.95 kPa (=relative air humidity of 70%) for 3 min. The average leaf temperature over the entire period was 25.2 ± 0.22°C for the constant VPD, 25.4 ± 0.31°C for the rapid VPD and 25.5 ± 0.28°C for the slow VPD treatment. The data are the means ± standard errors of six biological replicates.

Since we have found that the VPD fluctuation differing the amplitude would have different impacts on photosynthesis, we further examined the effects of the duration and the frequency of the VPD fluctuation on photosynthesis in the environmentally controlled chamber of portable photosynthesis system. Under constant VPD condition, transpiration rate, stomatal conductance, CO_2_ assimilation rate, and intercellular CO_2_ concentration were constant throughout the measurements (Figure 3). Both of rapid and slow VPD fluctuations induced the fluctuation of all the parameters. Transpiration rate, stomatal, conductance and intercellular CO_2_ concentration were maintained, while CO_2_ assimilation rate declined gradually under rapid VPD fluctuation. All the parameters declined gradually under slow VPD fluctuation. The larger decrease in CO_2_ assimilation rate was shown under slow VPD fluctuation than rapid VPD fluctuation during the measurement.

### Drastic VPD Fluctuation Caused Reduction in Plant Growth Compared to Moderate VPD Fluctuation

**Figure 4:**
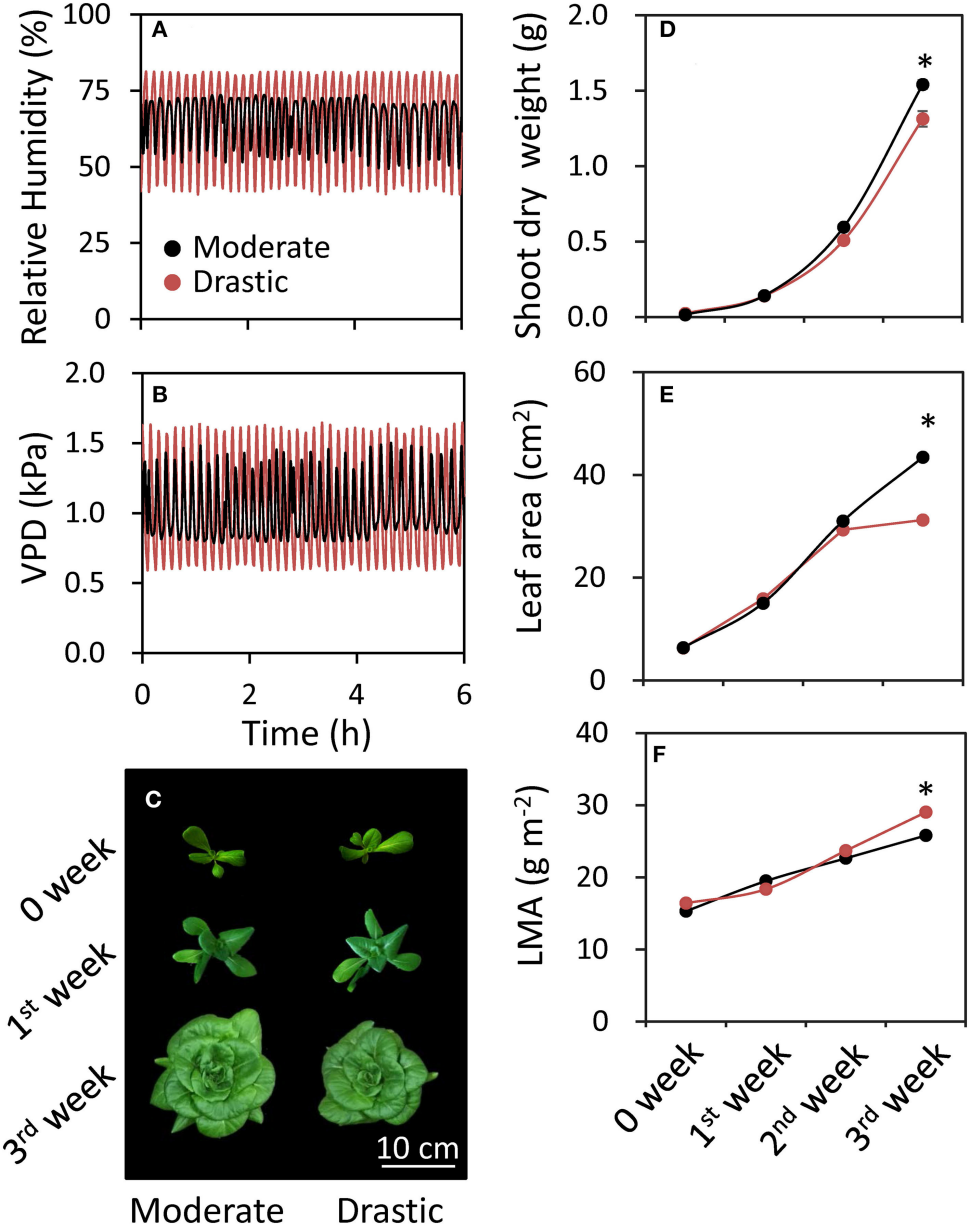
Responses of plant growth parameters to different fluctuating VPD conditions in lettuce. Plants were grown in the controlled growth chamber at a PPFD of 200 μmol photon m^−2^ s^−1^ under two different fluctuating VPD conditions: one is moderately fluctuating VPD condition, in which 7 min high VPD (1.32 kPa = relative air humidity of 55%) and 3 min low VPD (0.86 kPa = relative air humidity of 72%); or drastically fluctuating VPD condition, in which 6 min high VPD (1.63 kPa = relative air humidity of 42%) and 3 min low VPD (0.63 kPa = relative air humidity of 80%). Relative humidity **(A)** and VPD **(B)** during the experiment and a picture of lettuce plants in week 0, 1 and 3 **(C)** are shown. Shoot dry weight **(D)**, leaf area **(E)** and leaf mass per area (LMA) **(F)** of fully expanded leaves were analyzed every week until three weeks after the beginning of VPD treatments. The data are the means ± standard errors of six biological replicates. Significant differences between two different VPD conditions are examined by Student's *t*-test (*P < 0.05).

**Figure 5:**
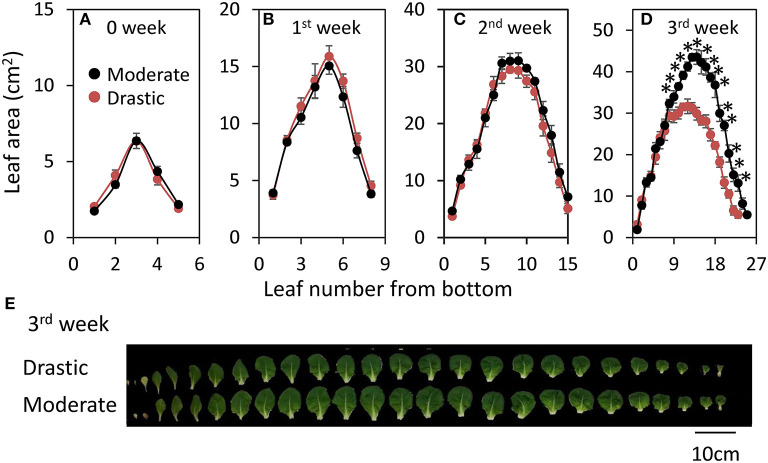
Responses of leaf number and leaf area to different fluctuating VPD conditions in lettuce. Plant growth conditions are similar to Figure 4. Leaf area was analyzed in all the leaves every week until three weeks after the beginning of VPD treatments **(A–D)**. Significant differences between two different VPD conditions are examined by Student's *t*-test (*P < 0.05). Pictures of each leaf was also summarized **(E)**.

Plant growth of lettuce was analyzed under moderately and drastically fluctuating VPD conditions (Figure 4). There was no clear difference of shoot dry weight, leaf area and LMA between two VPD conditions until two weeks after the beginning of the treatments (Figures 4C–F). On the other hand, the plants grown under drastic VPD fluctuation showed less shoot dry weight, leaf area and LMA than those under moderate VPD fluctuation at three weeks after the beginning of the treatments. The shoot dry weight and leaf area in plants grown under drastic VPD fluctuation was 15% and 29% lower than those under moderate VPD fluctuation, respectively. Although there was no difference of the leaf number per plant between two VPD conditions (24.7 ± 0.4 for moderate fluctuating VPD condition, 24.0 ± 0.4 for drastic fluctuating VPD condition; Figure 5), the plants grown under drastic VPD fluctuation showed smaller leaves with high LMA. The area of the leaves above the 9th leaf from the bottom were largely different between two VPD conditions at three weeks after the beginning of treatment (Figures 5D,E).

Although an air temperature was slightly different between two VPD conditions (Supplementary Figure 1), this difference was minimum and had no effect on CO_2_ assimilation rate (Supplementary Figure 2). There were no significant differences in leaf chlorophyll and anthocyanin content between two different VPD conditions (Table 1).

**Table 1:** Effect of VPD conditions on leaf chlorophyll and anthocyanin contents.

**Traits**	**Condition**	
	**Moderate**	**Drastic**
Chlorophyll (mmol m^−2^)	0.362 ± 0.009	0.376 ± 0.008
Anthocyanin (mmol m^−2^)	0.107 ± 0.024	0.113 ± 0.026

*Significant differences between two different VPD conditions are examined by Student's t-test. There were no significant differences in leaf chlorophyll and anthocyanin content between two different VPD conditions*.

## Discussion

### Fluctuating VPD Retarded Plant Growth via the Reductions in Leaf Area and Photosynthesis

VPD is considered to be one of the major environmental factors influencing stomatal conductance and photosynthesis (Raschke, 1970; Lange et al., 1971; Grange and Hand, 1987; Xu et al., 1991; Tinoco-Ojanguren and Pearcy, 1993; Bunce, 2006), as well as plant growth and development in crop and horticultural plants (Tibbitts, 1979; Grange and Hand, 1987; Bakker, 1991; Marsden et al., 1996; Leuschner, 2002; Codarin et al., 2006). Most of the previous studies focused on the effects of the averaged or steady-state VPD on plant growth. To our knowledge, there have been no report studying the effects of the VPD fluctuation on photosynthetic and growth performance in plants. The present study clearly showed that drastic VPD fluctuation in the environmentally controlled chamber of portable photosynthesis system declined stomatal conductance and thus CO_2_ assimilation rate (Figure 1), leading to photoinhibition (Figure 2; Yamori, 2016). Moreover, drastic VPD fluctuation for a long-term period resulted in a reduction of biomass production in lettuce (Figures 4, 5).

In general, during atmosphere water deficit, the decrease in stomatal conductance is the primary cause of the reduction of CO_2_ assimilation rate (e.g., Bunce, 1997, 2006). In the present study, drastic VPD fluctuation induced the declines in stomatal conductance, CO_2_ assimilation rate, ETR I and ETR II (Figures 1C,E,F). It would be considered that the drastic VPD fluctuation would cause stomatal closing, leading to simultaneous reductions in the CO_2_ assimilation rate and electron transport rate (Figure 1). This would result in an over-reduction of the plastoquinone pool (high 1-qL) and the long-term treatment of drastic VPD fluctuation caused severe photoinhibition (Figure 2). This was supported by the previous reports that the stomatal response to VPD is actively driven by an abscisic acid, ABA (Bauer et al., 2013) and that, at later stages with increasing severity, drought stress could lead to metabolic impairment including the declines in Rubisco activity (Parry et al., 2002). Thus, the increase in diffusive limitation via stomata and then biochemical limitation would be responsible for the decline in photosynthesis under the fluctuating VPD condition. Further studies would be required to quantitatively partition between stomatal and biochemical limitations with various time course owing to water severity.

Drastic VPD fluctuation declined CO_2_ assimilation rate and leaf area with no change in leaf number, resulting in the significant reduction of biomass production in lettuce (Figures 4, 5). There has been reported that VPD affects crop growth through not only a direct impact on CO_2_ assimilation rate and stomatal conductance but also on leaf size (Gisleroed and Nelson, 1989; Bakker, 1991). In addition, the reductions of leaf water potential and turgor are, for long, known to have a negative effect on leaf growth (Bradford and Hsiao, 1982; Kramer and Boyer, 1995) since even minimal reductions of leaf water potential or turgor can cause a significant reduction of leaf expansion (Acevedo et al., 1971; Dale, 1988; Hsiao et al., 1998; Alves and Setter, 2004) as well as cell number (Carins Murphy et al., 2014). Taken together, there is a strong relationship between water potential or turgor and leaf sizeas we have observed in leaf area in 3rd week after the biggining of the treatments (Figure 5).

### Importance of Fine-Regulation of VPD in Plant Growth Conditions

These days, greenhouse operations are moving toward controlling evaporative demand according to VPD from relative air humidity because this approach provides direct information about the driving force of transpiration and evaporation (Katul et al., 2009; Villarreal-Guerrero et al., 2012; du Plessis et al., 2015). The VPD regulation has been demonstrated as an efficient solution to maintain optimal ranges of temperature and relative air humidity simultaneously. Recent work showed that the VPD control via the fogging system improved plant productivity by enhancing the photosynthetic performance during the winter (Lu and Viljanen, 2009; Lu et al., 2015) and summer seasons (Zhang et al., 2015).

Although VPD control is important for the plant cultivation, daily and seasonal changes in VPD and solar radiation are large, and would have significant impacts on stomatal conductance, CO_2_ assimilation rate and plant growth (Myers et al., 1997; Prior et al., 1997; Hutley et al., 2000; Yamori, 2016). Even in greenhouse conditions, VPD fluctuates greatly during the day (Harmanto et al., 2005). In both fogging and fan-and-pad systems which have been commonly used for evaporative systems for cooling and humidifying greenhouses, VPD in greenhouses is commonly controlled by set points for VPD. As the set points are generally lower and upper VPD thresholds, the VPD control in the greenhouse is based on on/off regulation. As shown in the present study, fluctuating VPD consequently retarded plant growth (Figures 4, 5). Thus, VPD in the greenhouse should be controlled not by intermittent regulation but by continuous regulation. The effect of VPD on photosynthesis and plant growth would depend on the extent of fluctuations and the absolute value of VPD as well as other environmental conditions, including growth light intensity, CO_2_ concentration and wind velocity. The present study clearly showed that fine-regulation for stable environmental control in greenhouses could maintain the leaf expansion (Figure 5) and higher stomatal conductance and photosynthesis during the major part of the day (Figures 1–3), which would lead to the better plant growth and higher yield with high nutrition values in greenhouses (Figures 4, 5). The effect of VPD fluctuation level might affect processes underlying postharvest quality in lettuce (Chen et al., 2021). Further researches would be needed to optimize the continuous regulation of VPD for plant cultivation, with considering the mean VPD and the fluctuation range, in agricultural production.

## Data Availability Statement

The original contributions presented in the study are included in the article/Supplementary Material, further inquiries can be directed to the corresponding author.

## Author Contributions

TI, MS, MI, and WY conceived and designed the experiments. TI, MS, and WY performed the experiments and analyzed the data. QY, YM, KS, and WY prepared the manuscript, and all the members contributed extensively to its finalization.

## Conflict of Interest

TI, MS, and MI were employed by Fuji Silysia Chemical Co., Ltd. The remaining authors declare that the research was conducted in the absence of any commercial or financial relationships that could be construed as a potential conflict of interest.
