# Supplementary material for: Minimizing VPD Fluctuations Maintains Higher Stomatal Conductance and Photosynthesis, Resulting in Improvement of Plant Growth in Lettuce
Source: Front Plant Sci. 2021 Apr 1;12:646144. doi: 10.3389/fpls.2021.646144 (PMC8049605; doi:10.3389/fpls.2021.646144)

Minimizing VPD fluctuations maintains higher stomatal conductance and photosynthesis, resulting in improvement of plant growth in lettuce

Takayasu Inoue[1], Motoo Sunaga[1], Mutsuhiro Ito[2], Qu Yuchen[3], Yoriko Matsushima[3], Kazuma Sakoda[3] and Wataru Yamori[3*]

[1]Fuji Chemical Co., LTD
[2]Fuji Silysia Chemical Co., LTD
[3]Institute for Sustainable Agro-Ecosystem Services, The University of Tokyo, Nishitokyo, Japan

***Corresponding author:**
Wataru Yamori
yamori@g.ecc.u-tokyo.ac.jp

Supplemental Fig. 1.
Fluctuation of air temperature observed in two different fluctuating VPD conditions. An air temperature in the growth chamber was monitored while plants were grown under two different fluctuating VPD conditions, as shown in Figure 4.

Supplemental Fig. 2.
Responses of photosynthetic parameters to two difference temperature conditions in lettuce. A CO_2 assimilation rate was measured under constant temperature of 25˚C or fluctuating temperature alternating 25˚C and 23˚C every 10 min which is similar to the condition observed in supplemental Figure 1.

Supplemental Figure 1

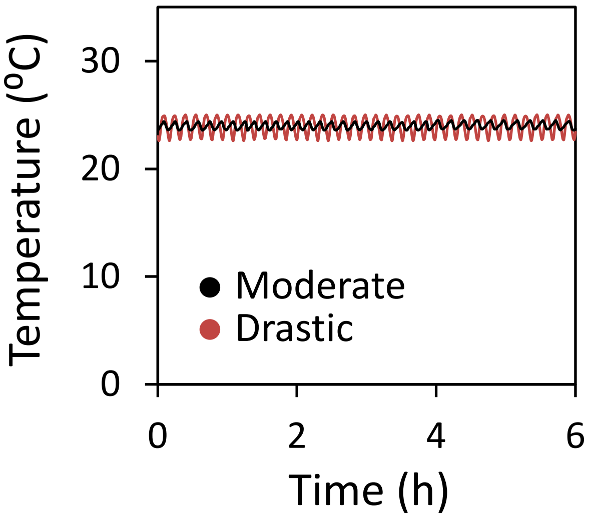

Supplemental Figure 2

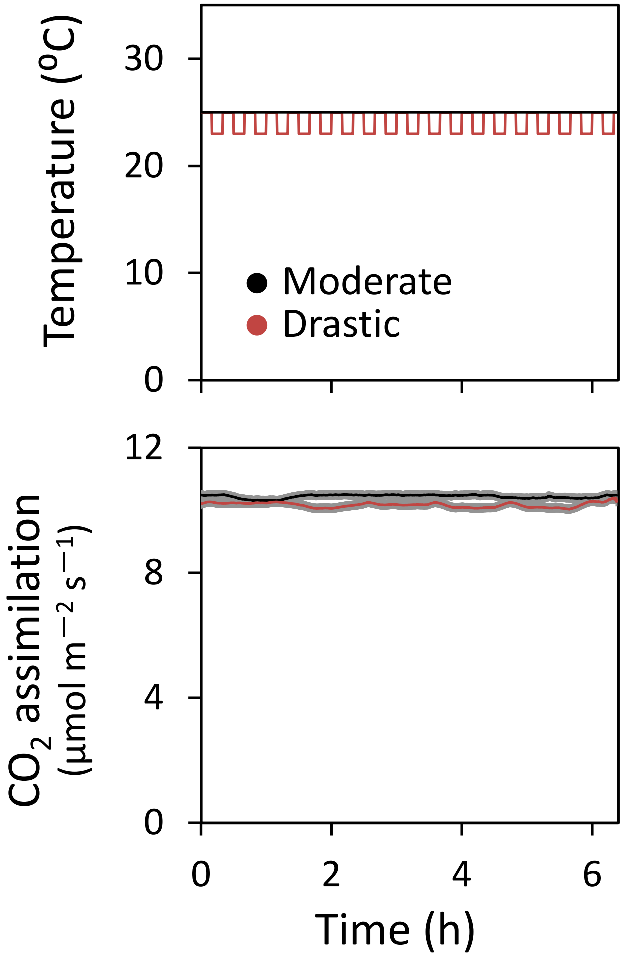

Supplement: Supplementary file 1 [file Data_Sheet_1.PDF]
